# Inhibition of colorectal cancer cell growth by downregulation of M2-PK and reduction of aerobic glycolysis by clove active ingredients

**DOI:** 10.3389/fphar.2025.1552486

**Published:** 2025-04-16

**Authors:** Lin Liu, Gang Xing, Xiaoyi Guo, Hui Chen, Jian Li, Jian Wang, Yaling Li, Gang Liang, Minghua Liu

**Affiliations:** ^1^ School of Pharmacy, Southwest Medical University, Luzhou, China; ^2^ Drug Dispending Department, The Third Hospital of Mianyang, Sichuan Mental Health Center, Mianyang, China; ^3^ Pharmacy Department, Affiliated Traditional Chinese Medicine Hospital of Southwest Medical University, Luzhou, China; ^4^ Discipline Construction Office, Affiliated Traditional Chinese Medicine Hospital of Southwest Medical University, Luzhou, China; ^5^ Pharmacy Department, Affiliated Hospital of Southwest Medical University, Luzhou, China

**Keywords:** active fraction from clove, M2-type pyruvate kinase, aerobic glycolysis, anti-colon cancer, cyclin D1

## Abstract

Exploring the anti-tumor molecular mechanisms of traditional Chinese medicines has become an important strategy to develop novel anti-tumor drugs in the clinic. Several pharmacological studies have reported the antioxidant, antibacterial, anti-inflammatory, and anti-tumor effects of clove. Previously, we have shown that the active fraction from clove (AFC) can inhibit the growth of tumor cells, particularly colon cancer cells, *in vitro*. However, the mechanism of action regarding the anti-colon cancer activity of AFC, especially in aerobic glycolysis, has not been adequately investigated. In this study, we found that AFC significantly inhibited the growth of five types of colon cancer cells, downregulated the mRNA and protein levels of M2-type pyruvate kinase (PKM2), and reduced aerobic glycolysis capacity. Transfection of PKM2-siRNA mimicked the inhibitory effects of AFC on aerobic glycolysis in colon cancer cells. Furthermore, the highly expressed, tumor-specific targets c-myc and cyclin D1 in cells were also found to be downregulated following the action of AFC. In the HCT116 cell xenograft nude mice models, the results after AFC administration were consistent with those of the cellular experiments, while AFC caused less liver injury and weight loss than the conventional chemotherapeutic agent 5- fluorouracil (5-FU). In conclusion, AFC inhibits colon cancer growth by downregulating PKM2 to inhibit aerobic glycolysis and reduce the tumor-specific high expression of c-myc and cyclin D1. Future work should explore how it downregulates pyruvate kinase (PK) in the first place, along with the intrinsic mechanism between the downregulation of PKM2 and the downregulation of c-myc.

## 1 Introduction

Colorectal cancer (CRC) accounts for 10% of all new cancer diagnoses and cancer-related deaths worldwide each year (Ahmad et al., 2021; Brenner et al., 2014), and its global incidence is expected to increase as populations age and adopt Western lifestyles (Dekker et al., 2019). Surgery can prolong the survival of patients with non-metastatic colorectal cancer, while those ineligible for surgery can benefit from chemotherapeutic agents such as fluorouracil, oxaliplatin, and irinotecan (Benson et al., 2018). Resistance to these drugs and their adverse effects (Kuipers et al., 2015; Morton et al., 2023) have led many to prefer traditional Chinese medicine, which can be effective but whose mechanisms remain unclear (Wang et al., 2021), which hinders its wider use and rational optimization.

The Warburg effect, in which tumor cells, regardless of the presence or absence of oxygen, rely primarily on glycolysis for metabolism, consume large amounts of glucose, and rapidly produce lactate and ATP, is an important difference in the metabolic profile of tumor cells and normal cells (Vaupel and Multhoff, 2021). Cancer cells metabolize glucose much more efficiently than normal cells, so their glucose uptake capacity exceeds that of normal cells and promotes energy supply for their unlimited proliferation. In addition, massive intermediate metabolites of aerobic glycolysis can further promote the metabolism of pentose phosphate and other bypass routes for energy supply. A large amount of metabolic end products, such as lactic acid, are secreted into the extracellular area to form an acidified tumor microenvironment, which again enhances the invasiveness and metastasis of the tumor.

Pyruvate kinase (PK), the important rate-limiting enzyme for the final irreversible step of glycolysis, has four isozymes (M1, M2, L, and R) (Palsson-McDermott et al., 2015). During tumor formation, intracellular M2-type pyruvate kinase (PKM2) is abundantly expressed in place of the original tissue-specific pyruvate kinase isoform until it becomes the predominant isoform (Chaneton and Gottlieb, 2012). In addition to its role in regulating metabolism, PKM2 acts as an upstream regulatory protein in the nucleus and activates c-myc and cyclin D1 gene transcription through histone 3 phosphorylation at the threonine 11 residue (H3-T11), which further promotes cell cycle progression and cell proliferation (Venneti and Thompson, 2013; Yang et al., 2012; Zheng et al., 2020). Moreover, c-myc and cyclin D1 are typical tumor-associated genes. C-myc is the transcriptional regulator involved in regulating the expression of approximately 15% of the genes in the human genome (Patel et al., 2004). Intracellularly, c-myc is a highly unstable protein, is closely related to tumorigenesis and progression, and is highly expressed in colorectal cancer (Moradifard et al., 2022; Wang et al., 2017). The other regulator, cyclin D1, plays a positive regulatory role in the G1/S transition, a key restriction point in the cell cycle, and takes an important role in the genesis and progression of a variety of tumors (Wang et al., 2023). Overexpression of cyclin D1 may cause cycle disorders, DNA damage, and mutant cell replication and division, leading to tumor development. Therefore, cyclin D1 has become a well-recognized proto-oncogene, and its overexpression causes 55% of the total number of colorectal cancers (Musgrove et al., 2011).

Clove, the dried and scented buds of the Myrtaceae family plants *Eugenia caryophyllata Thunb.* or *Syzygium aromaticum* (L.) (Merr. and L.M.Perry.), also known as an ornamental and spice plant, is stipulated in Chinese Pharmacopoeia (Commission, 2020). This traditional Chinese food-homology herb, containing β‐caryophyllene and flavonoids, such as eugenin, eugenol, and oleanolic acid (Cortés-Rojas et al., 2014), is recognized for its bioactive compounds with various pharmacological effects, such as anti-oxidant, anti-bacterial, anti-inflammatory, and anti-tumor activities (Zari et al., 2021; Radünz et al., 2019; Chniguir et al., 2019).β‐caryophyllene reduces inflammatory mediators to improve metabolic and neurological chronic oxidative stress disorders (Scandiffio et al., 2020). The flavonoid eugenin can stimulate human peripheral blood T-cell activation (Chang et al., 2003). Eugenol possesses anticancer potential in terms of anti-metastatic, anti-proliferative, anti-angiogenic, cell cycle arrest, apoptotic, and autophagic actions (Begum et al., 2022). Oleanolic acid has anticancer, anti-inflammatory, hepatoprotective, gastroprotective, hypolipidemic, and anti-atherosclerotic activities (Castellano et al., 2022). We and others have previously shown that the active fraction from clove (AFC, containing mainly oleanolic acid and eugenol), obtained through 95% ethanol extraction of the dried flower buds (Liu et al., 2018), can significantly inhibit the growth of lung, breast, liver, pancreatic, ovarian, and cervical cancer cells *in vitro* (Wei et al., 2012; Liu et al., 2014a; Liu et al., 2014b; Liu et al., 2014c; Liu H. et al., 2014). Oleanolic acid induces apoptosis in colon cancer cells by inhibiting signaling involving PI3 kinase, Akt, and mTOR (Zhao et al., 2021). In addition, oleanolic acid inhibits aerobic glycolysis in human prostate and breast cancer cells (Liu et al., 2014e). However, AFC’s effects against colon cancer appear to be particularly stronger in nude mice bearing human colon cancer HT-29 xenografts compared to oleanolic acid (Liu H. et al., 2014).

Aerobic glycolysis is a biological process unique to tumor cells, but there is a lack of drugs specifically targeting this process (Fukushi et al., 2022) and even less research on active drugs in traditional Chinese medicine. PKM2 is a potentially valuable marker for tumor diagnosis and for the prognostic evaluation of its therapeutic efficacy, and it may also be an important target for anti-tumor drugs. Given the background of the abovementioned previous studies, we investigated whether the mechanism of action of AFC on colon cancer involves the inhibition of aerobic glycolysis by affecting PKM2.

Here, we provide evidence, *in vitro* and *in vivo,* that AFC downregulates PKM2 to inhibit aerobic glycolysis in colon cancer. In addition, we found that it downregulates c-myc and cyclin D1, which are normally upregulated in colon cancer to drive tumor proliferation. Our findings suggest that AFC exerts its anti-tumor effects through both metabolic and non-metabolic pathways. The results may provide a theoretical basis for the translation of AFC into clinical antitumor drugs and serve as a reference for the exploration of new key therapeutic targets within the aerobic glycolysis and non-metabolic pathways in colon cancer.

## 2 Materials and methods

### 2.1 Preparation of AFC

Clove is derived from the dried flower buds of the plants *E. caryophyllata Thunb.* or *S. aromaticum* (L.) Merr. and L.M.Perry in the Myrtaceae family. AFC, containing mainly eugenol and oleanolic acid, was obtained from dried clove powder by using the method described in our previous research (Liu et al., 2018). Analyses of all components of AFC have been reconfirmed in previous studies (Li et al., 2019). In subsequent experiments, we dissolved AFC in a serum-free medium using ultrasonication to prepare a drug master stock at a concentration of 4 mg/mL.

### 2.2 Reagents and antibodies

Dulbecco’s modified Eagle medium (DMEM) and RPMI-1640 were purchased from Gibco (Thermo Fisher Scientific, Waltham, MA, United States of America); the heat-inactivated fetal bovine serum was purchased from EVERY GREEN (Zhejiang, China); the penicillin–streptomycin solution was purchased from Beyotime (Shanghai, China); the cell counting kit-8 (CCK-8), from AbMole (Shanghai, China); assay kits for glucose and lactic acid, from Nanjing Jiancheng Bioengineering Institute (Nanjing, Jiangsu, China); assay kits for pyruvate and pyruvate kinase, from Beijing Solarbio Science and Technology (Beijing, China). A kit for reverse transcription and the FastFire Premix (SYBR Green) kit for quantitative PCR were purchased from Tiangen (Beijing, China). Primary antibodies against PKM2 (cat. no. 60268-1-Ig), cyclin D1 (cat. no. 60186-1-Ig), c-myc (cat. no. 67447-1-Ig), or β-actin (cat. no. 66009-1-Ig) and the horseradish peroxidase-labeled goat anti-mouse IgG (H + L) antibody (cat. no. SA00001-1) were purchased from Proteintech (Hubei, China). The primary antibody against Ki67 (cat. no. GB121141) and horseradish peroxidase-conjugated goat anti-mouse IgG (H + L) (cat. no. GB23301) were purchased from Servicebio (Wuhan, China).

### 2.3 Cell culture and treatments

The following five human colorectal cancer cell lines were purchased from the American Type Culture Collection (Manassas, VA, United States of America): HCT-116, SW620, Caco-2, HT29, and LoVo. Cells were cultured at 37°C in an atmosphere of 5% CO_2_ in DMEM or RPMI-1640 medium containing 10% heat-inactivated fetal bovine serum, 100 U/mL penicillin, and 100 mg/mL streptomycin. The medium was replaced every 2–3 days. In culture experiments, AFC was dissolved to a concentration of 4 mg/mL in the serum-free medium through sonication and then diluted with the culture medium to concentrations of 25, 50, 100, 200, or 400 μg/mL. Cultures were exposed to these dilutions for 24, 48, or 72 h. Control cultures were treated with the medium without AFC. All cell lines were identified by short tandem repeat analysis and routinely tested negative for pathogens and *Mycoplasma*.

### 2.4 Anti-cell proliferation assay

#### 2.4.1 CCK8 assay

Cells at 90% confluence were seeded into 96-well plates at the density of 5 × 10^3^ cells/well, incubated for 24 h, and then exposed for 24, 48, or 72 h to different concentrations of AFC in the medium, or they were exposed to the medium without AFC as a negative control. After each time point, diluted CCK8 reagent (10 μL CCK8 + 90 μL serum-free medium) was added to each well, the wells were incubated at 37°C for 1 h, and the optical density at a wavelength of 450 nm was measured using the Cytation 3 microplate reader (Bio-tek, VT Laboratories, United States of America). In addition to the experimental and control wells, “blank” wells containing only the medium and CCK8 were processed in parallel. Cell survival was calculated as [(OD _experimental_ − OD _blank_)/(OD _control_ − OD _blank_)] × 100%. Growth inhibition was calculated as [(OD _control_ − OD _experimental_)/(OD _control_ − OD _blank_)] × 100%. Data for all samples were collected from three independent experiments.

#### 2.4.2 Colony formation assay

HCT116 or LoVo cells were seeded into six-well plates (500 cells/well), cultured for 24 h, and then exposed to AFC at 25, 50, 100, or 200 mg/mL; the group that only received an equal amount of the serum-free medium was regarded as the negative control group. Plates were incubated for 48 h, and then, the culture medium was replaced and cultured for 12 days, during which the medium was changed every 4 days. Finally, cells were fixed with 4% paraformaldehyde (Servicebio, Wuhan, China), stained with 1% crystalline violet (Beyotime, Beijing, China) for 10 min at room temperature, and counted using ImageJ (version 1.8.0; US National Institutes of Health, Bethesda, MD, United States of America).

### 2.5 Aerobic glycolysis capacity assay

#### 2.5.1 Glucose consumption

HCT116 or LoVo cells were inoculated in six-well plates (4 × 105 cells/well), cultured for 24 h, and then treated with AFC or the medium without AFC for 48 h. The culture medium was collected and centrifuged at 1,000 g for 10 min; then, the supernatant was assayed for glucose levels using a commercial kit. Samples were incubated at 37°C for 10 min, and then the optical density at 505 nm was measured. We converted the optical density readings to concentrations according to the kit instructions. Data for all samples were collected from three independent experiments.

#### 2.5.2 Lactate production

Cells were cultured and treated with AFC, as described in the *Glucose consumption* section; then, the culture medium was collected, diluted two-fold with distilled water, and processed using a commercial kit to assay lactate concentration. The optical density at 530 nm was measured. We converted the optical density readings to concentrations based on the instructions for the kit. Data for all samples were collected from three independent experiments.

#### 2.5.3 Pyruvate production

Cells were cultured and treated with AFC as described in the *Glucose consumption* section; then, the cells were harvested, lysed by ultrasonication and allowed to stand at 0°C for 30 min, and centrifuged at 8,000 g for 10 min at room temperature. The supernatant was processed using a commercial kit to assay pyruvate concentrations based on the optical density at a wavelength of 520 nm. Pyruvate production was calculated according to the operating manual instructions. Data for all samples were collected from three independent experiments.

#### 2.5.4 Pyruvate kinase activity

Cells were cultured at a density of 2 × 10^6^ in 60-mm cell-culture dishes and treated with AFC, as described in the *Glucose consumption* section; then, cells were harvested, lysed by ultrasonication, and centrifuged at 8,000 g at 4°C for 10 min. The supernatant was processed using a commercial kit to determine the optical density A1 at a wavelength of 340 nm after 20 s of the reaction at 37°C and optical density A2 after 2 min of the reaction. Pyruvate kinase activity was calculated according to the operating manual instructions. Data for all samples were collected from three independent experiments.

### 2.6 Molecular docking prediction

The 3D structures of the constituent compounds of AFC were downloaded from PubChem (https://pubchem.ncbi.nlm.nih.gov/) in the SDF format, and the three targets involved in the scientific hypothesis of this study, PKM2, c-myc, and cyclin D1, were downloaded from the Protein Data Bank (https://www.rcsb.org/) in the PDB format. Compounds and target files were then uploaded to DockThor (https://dockthor.lncc.br/v2/) for online molecular docking, and software Chimera X was used to visualize the resulting conformational maps.

### 2.7 Western blotting

Cells were cultured and treated with AFC, as described in the *Glucose consumption* section; then, the cells were washed twice with pre-cooled phosphate-buffered saline (PBS) at 4°C, lysed with RIPA lysis buffer (Beyotime, Shanghai, China) containing 1% phenylmethyl sulfonyl fluoride (Solarbio, Beijing, China) and 1% protease inhibitor cocktail (Epizyme, Shanghai, China) on ice, harvested, and centrifuged at 12,000 g for 20 min at 4°C. The supernatant was assayed using the BCA protein assay (KeyGEN BioTECH, Nanjing, Jiangsu, China), and equal amounts of protein in each sample (15 μg) were fractionated using 10% sodium dodecyl sulfate–polyacrylamide gel electrophoresis and then electrotransferred onto polyvinylidene difluoride membranes (0.45 mm, Millipore, NY, United States of America). Membranes were blocked for 15 min at room temperature with protein-free fast blocking solution (Epizyme, Shanghai, China) that had been diluted to 1X with Tris-buffered saline containing Tween-20 (TBST) and then incubated overnight at 4°C with primary antibodies (diluted 1:5,000) against PKM2, cyclin D1, c-myc, or β-actin. Membranes were washed three times with TBST and then incubated at room temperature for 1 h with the horseradish peroxidase-coupled goat anti-mouse secondary antibody (diluted 1:5,000). Antibody binding was visualized using the ECL chemiluminescence reagent (Affinity, Jiangsu, China), imaged using a Bio-Rad imaging system (Bio-Rad, version 2.0.0.27, United States of America), and quantified using ImageJ (version 1.8.0, US National Institutes of Health, Bethesda, MD, United States of America). Protein levels were normalized to those of β-actin. Data for all samples were collected from three independent experiments.

### 2.8 Reverse transcription quantitative polymerase chain reaction

Cells were cultured and treated with AFC, as described in the *Glucose consumption* section; then, cells were harvested, and total cellular RNA was extracted using TRIzol (Invitrogen, Thermo Fisher Scientific, Inc.), reverse-transcribed into cDNA using a commercial kit (Tiangen, Beijing, China), and subjected to quantitative PCR using the primers described in Table 1, FastFire qPCR Premix (Tiangen, Beijing, China), and the CFX Connect Real-Time system [BioRad, version 1.0 (4.0.2325.0418), United States of America]. PCR involved pre-denaturation at 95°C for 1 min, followed by 40 cycles of denaturation at 95°C for 5 s, annealing, and extension at 60°C for 15 s. Levels of mRNAs were determined using the 2^−ΔΔCt^ method and normalized to those of GAPDH mRNA. Data for all samples were collected from three independent experiments.

**TABLE 1:** Primers used for quantitative PCR.

Target gene	Primer direction	Primer sequence (5′–3′)	Accession no.
Cyclin D1	F	GCT​GCG​AAG​TGG​AAA​CCA​TC	NM_053056.3
	R	CCT​CCT​TCT​GCA​CAC​ATT​TGA​A	
c-myc	F	GGC​TCC​TGG​CAA​AAG​GTC​A	NM_002467.6
	R	CTG​CGT​AGT​TGT​GCT​GAT​GT	
PKM2	F	ATG​TCG​AAG​CCC​CAT​AGT​GAA	NM_002654.6
	R	TGG​GTG​GTG​AAT​CAA​TGT​CCA	
GAPDH	F	GGA​GCG​AGA​TCC​CTC​CAA​AAT	NM_001256799.3
	R	GGC​TGT​TGT​CAT​ACT​TCT​CAT​GG	

F, forward; PKM2, M2-type pyruvate kinase; R, reverse.

### 2.9 Knockdown of PKM2

In brief, HCT116 or LoVo cells were inoculated in 96-well plates or six-well plates at appropriate densities. When cells reached 80% confluence, they were transfected with short interfering RNAs (siRNAs) targeting PKM2 (GenePharma, GenBank Accession no. NM_002654.6, Shanghai, China) (sense 5′-GGC​UGG​ACU​ACA​AGA​ACA​UTT-3’; antisense 5′-AUG​UUC​UUG​UAG​UCC​AGC​CTT-3′) or negative control RNA (sense 5′-UUC​UCC​GAA​CGU​GUC​ACG​UTT-3’; antisense 5′-ACG​UGA​CAC​GUU​CGG​AGA​ATT-3′) using Lipofectamine 3000 (Invitrogen, Thermo Fisher Scientific, Inc.), according to the manufacturer’s instructions. The siRNAs were mixed with Lipofectamine in fresh serum-free medium, incubated for 15 min at room temperature in the dark, and added at a concentration of 0.2 μg/well to 96-well plates or 5 μg/well to six-well plates for 6 h. Then, the transfection medium was replaced with fresh medium with or without AFC, and cultures were incubated for 48 h. Cells were analyzed for viability, as described in the *Cell viability* section, and for aerobic glycolysis, as described in the *Aerobic glycolysis capacity assay* section*.*

### 2.10 Antitumor activity *in vivo*

A total of 30 male BALB/c nude mice (Gempharmatech Co., Ltd, Sichuan, China), 4–5 weeks old and weighing 18–22 g, were housed (22°C and 40%–70% humidity, with a 12-h light/dark cycle and *ad libitum* availability of food and water) at the Experimental Animal Center of Southwest Medical University and subcutaneously inoculated with HCT116 cells (1.5 × 10^6^ in 100 μL) in the right axilla. When the tumor volume reached approximately 50 mm^3^ (approximately a week), the mice were randomly divided into five groups of six animals each: the control group received saline, the positive control group received 5-fluorouracil (5-FU) at 25 mg/kg, and three other groups received AFC at doses of 25, 50, or 100 mg/kg. All treatments were delivered intraperitoneally in a volume of 100 μL every other day. The tumor volume was measured daily using the following formula: [0.5 × (long diameter) × (short diameter)] (Brenner et al., 2014). The tumor inhibition rate was measured as [(mean tumor mass of the control group − mean tumor mass of the treatment group)/mean tumor mass of the control group] × 100%. Mouse body weight was also measured daily. After 10 administrations, animals were anesthetized in the R500IE animal anesthesia machine (3.5% isoflurane, 3.5 L/min gas flow rate, RWD, Guangdong, China) and subsequently sacrificed by cervical dislocation; tumors were excised, weighed, fixed with 10% formaldehyde, embedded in paraffin, and cut into 4-μm sections. Sections were dehydrated using xylene and ethanol at 37°C, and then, peroxidase was blocked with 3% H_2_O_2_ for 10 min. Blocked with 3% bovine serum albumin (Servicebio, Wuhan, China) for 20 min at room temperature, the sections were incubated overnight at 4°C with PKM2 (diluted 1:100) or Ki67 (diluted 1:100). Then, the horseradish peroxidase-conjugated goat anti-mouse IgG antibody (H + L) (diluted 1:100) was incubated at 37°C for 30 min. DAB chromogenic kits (ZSGB-BIO, Beijing, China) were used to visualize the signals. Depending on the experiment, the tissue was also stained with hematoxylin (dyeing for 15 min) and eosin (dyeing for 5 min) at room temperature. All sections were examined histologically in the digital pathology scanner (KF-PRO-002, KFBIO, Zhejiang, China).

The animal study was reviewed and approved by the Animal Ethics Committee of Southwest Medical University (approval swmu20230080).

All animal experiments complied with the ARRIVE guidelines were carried out in accordance with the United Kingdom. Animals (Scientific Procedures) Act, 1986 and associated guidelines, EU Directive 2010/63/EU for animal experiments.

### 2.11 Statistical analysis

All experimental data were analyzed using GraphPrism 8.0 (San Diego, CA, United States of America) and expressed as mean ± standard deviation (SD). Differences between two groups were assessed for significance using one-way ANOVA and Tukey’s test, while differences among at least three groups were assessed using two-way ANOVA.

## 3 Results

### 3.1 AFC inhibits the growth of various colon cancer cell lines

**FIGURE 1:**
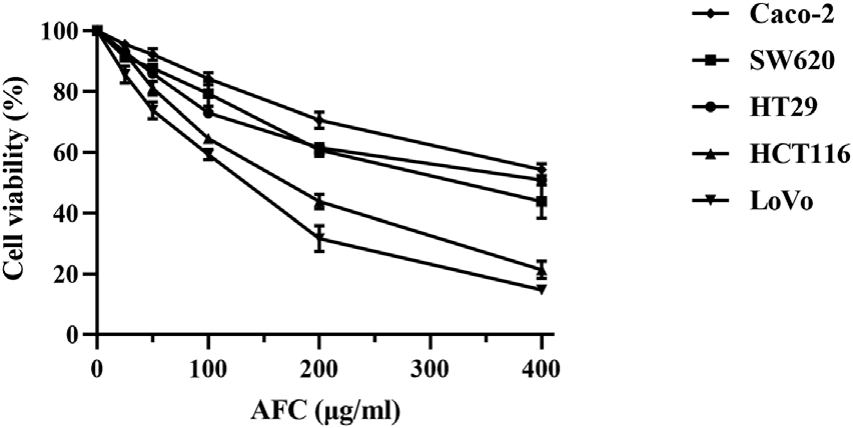
Viability of different human colon cancer cell lines after exposure for 48 h to different concentrations of AFC. Data are mean ± SD of three independent experiments.

AFC significantly inhibited the growth of five colon cancer cell lines: HCT-116, SW620, Caco-2, HT29, and LoVo (Figure 1, Table 2).

**TABLE 2:** IC_50_ values for AFC against five human colon cancer cell lines.

Cell line	IC_50_(μg/mL)
Caco-2	464.00 ± 21.13
HT29	380.80 ± 8.96
SW620	318.33 ± 28.87
HCT116	155.30 ± 12.72
LoVo	116.20 ± 4.10

Values are expressed as mean ± SD of three independent experiments.

**FIGURE 2:**
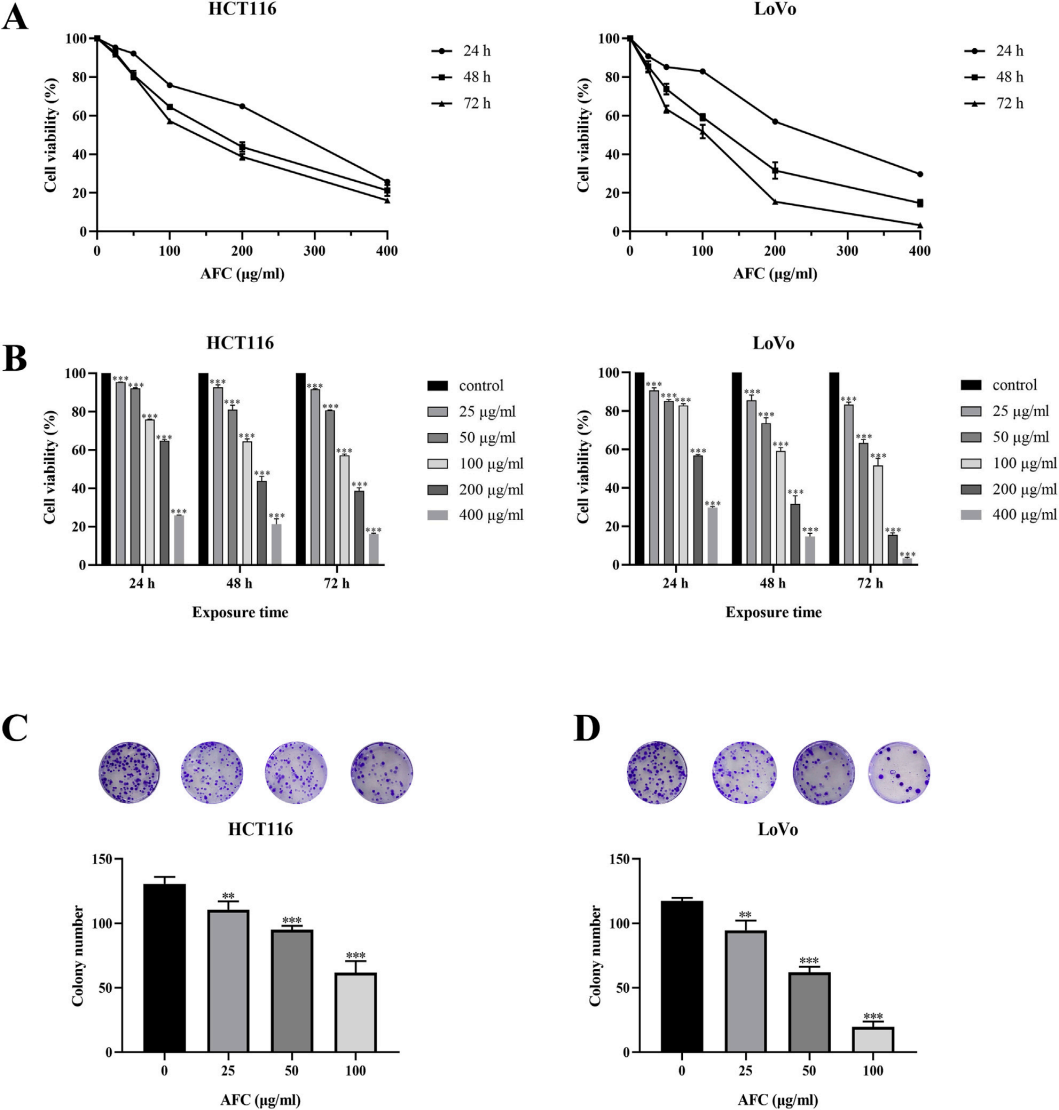
Effects of AFC on **(A, B)** viability and **(C, D)** colony formation by human colon cancer cell lines HCT-116 and LoVo. The assays in panels **(C, D)** were conducted after 48-h exposure. Data are expressed as mean ± SD of three independent experiments. *P < 0.05, **P < 0.01, and ***P < 0.001 vs vehicle control.

We focused on HCT116 and LoVo cells in subsequent experiments because these two cell lines had the most significant growth inhibitory effect after 24, 48, and 72 h of AFC treatment among the five cell lines (Table 2), and we found that the growth of both cell lines was inhibited in a concentration- and time-dependent manner (Figures 2A, B). Similar results were observed in a colony formation assay (Figures 2C, D).

### 3.2 AFC inhibits aerobic glycolysis in colon cancer cells

**FIGURE 3:**
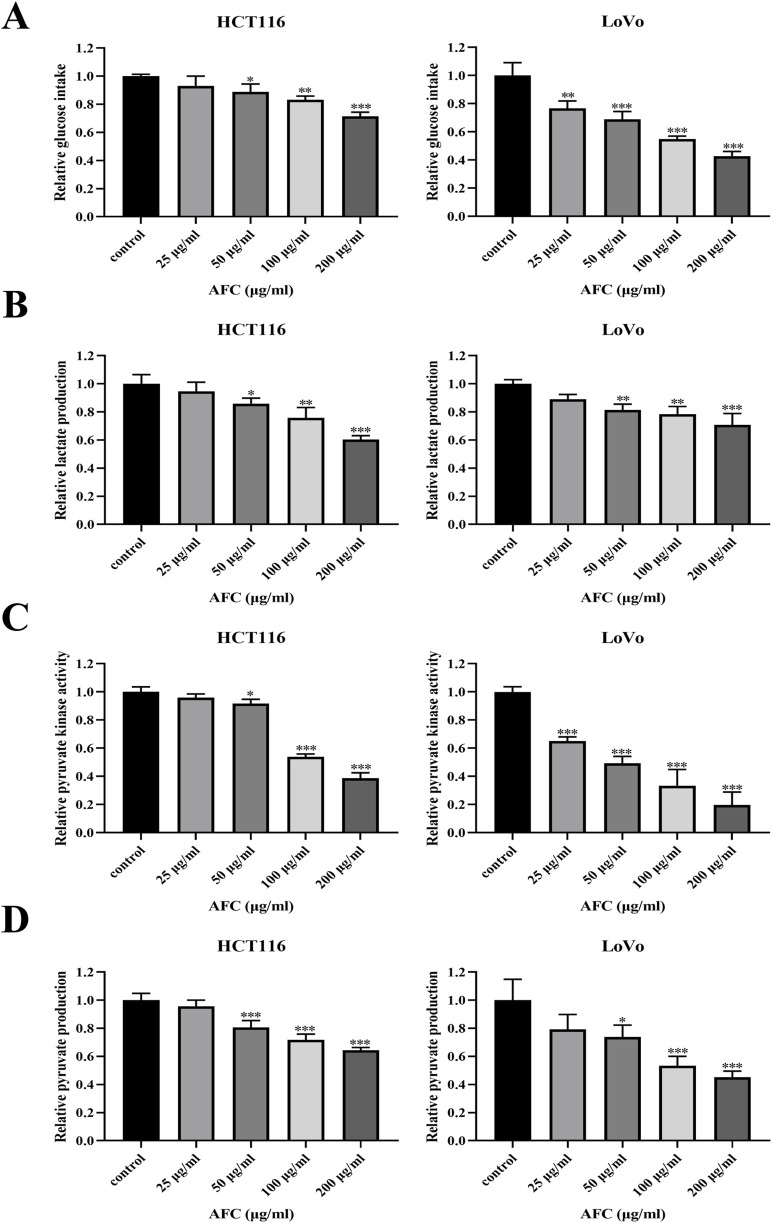
Effects of AFC on **(A)** glucose intake, **(B)** lactate production, **(C)** pyruvate kinase activity, and **(D)** pyruvate production in the human colon cancer cell lines HCT116 and LoVo. Results are shown relative to the value in the corresponding control group, which was defined as 1.0. Data are expressed as mean ± SD of three independent experiments. *P < 0.05, **P < 0.01, and ***P < 0.001 vs vehicle control.

Treating HCT116 and LoVo cells with AFC significantly reduced glucose uptake (Figure 3A), lactate production (Figure 3B), pyruvate kinase activity (Figure 3C), and pyruvate production (Figure 3D) in a dose-dependent manner.

### 3.3 AFC has good binding activity with PKM2 and related targets

**FIGURE 4:**
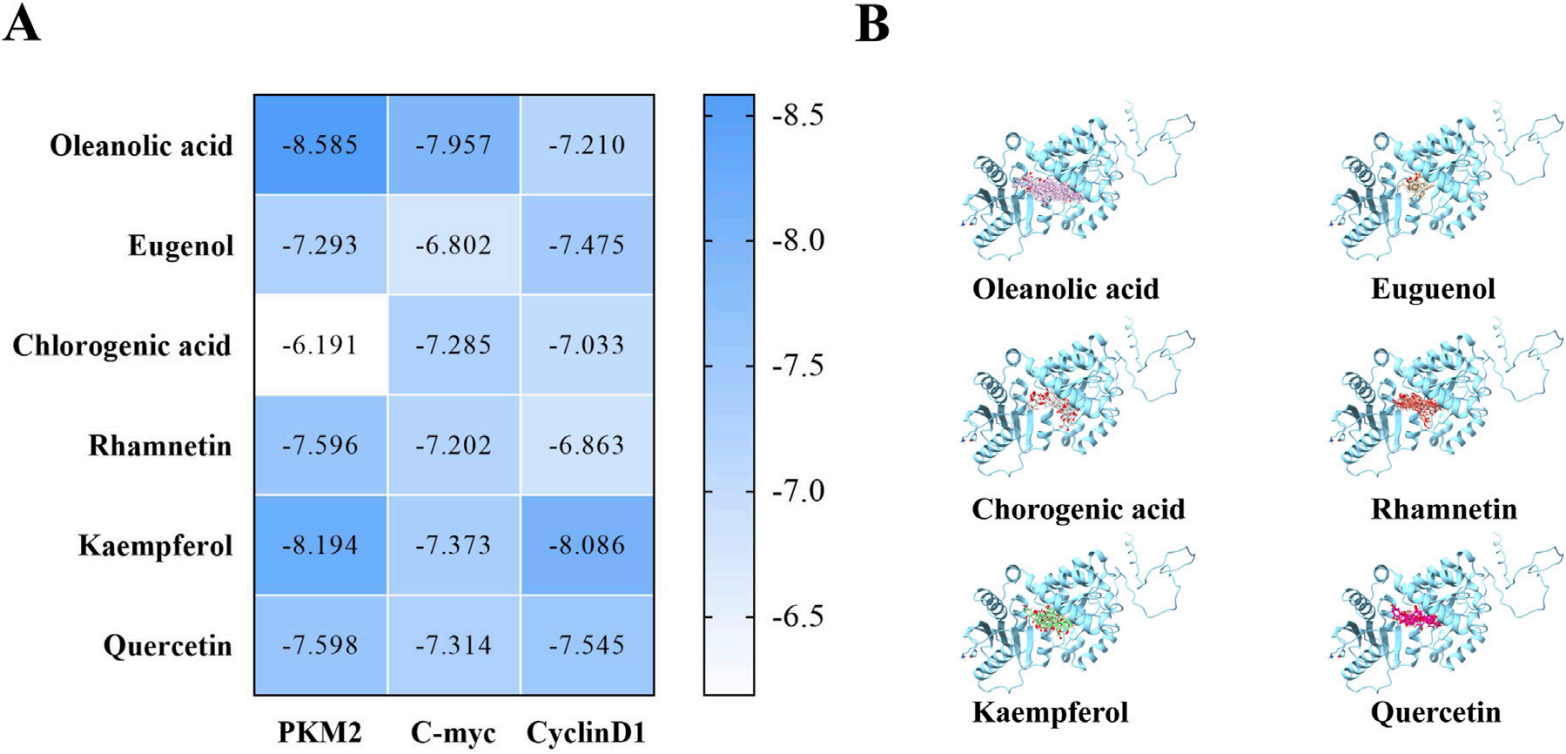
Molecular docking prediction results. **(A)** Docking scores between protein targets and each active monomer of AFC **(B)** Docking conformation diagram of the PKM2 protein with each active molecule of AFC.

To assess the potential mechanism of action between AFC and aerobic glycolysis in colon cancer cells, we used DOCK docking software to predict the binding activities of six active components of AFC (oleanolic acid, eugenol, chlorogenic acid, rhamnetin, kaempferol, and quercetin) with the metabolic target PKM2, and we also investigated the binding activities of the components with the typical high-expression targets of colon cancer, c-myc and cyclin D1. The predicted results revealed that the three targets, especially PKM2, had strong binding activities with all active components, especially the two most abundant major components of AFC, oleanolic acid and eugenol. Docking scores and docking simulation plots for all components are presented in Figure 4.

### 3.4 AFC inhibits aerobic glycolysis and proliferation of colon cancer cells by downregulating PKM2

**FIGURE 5:**
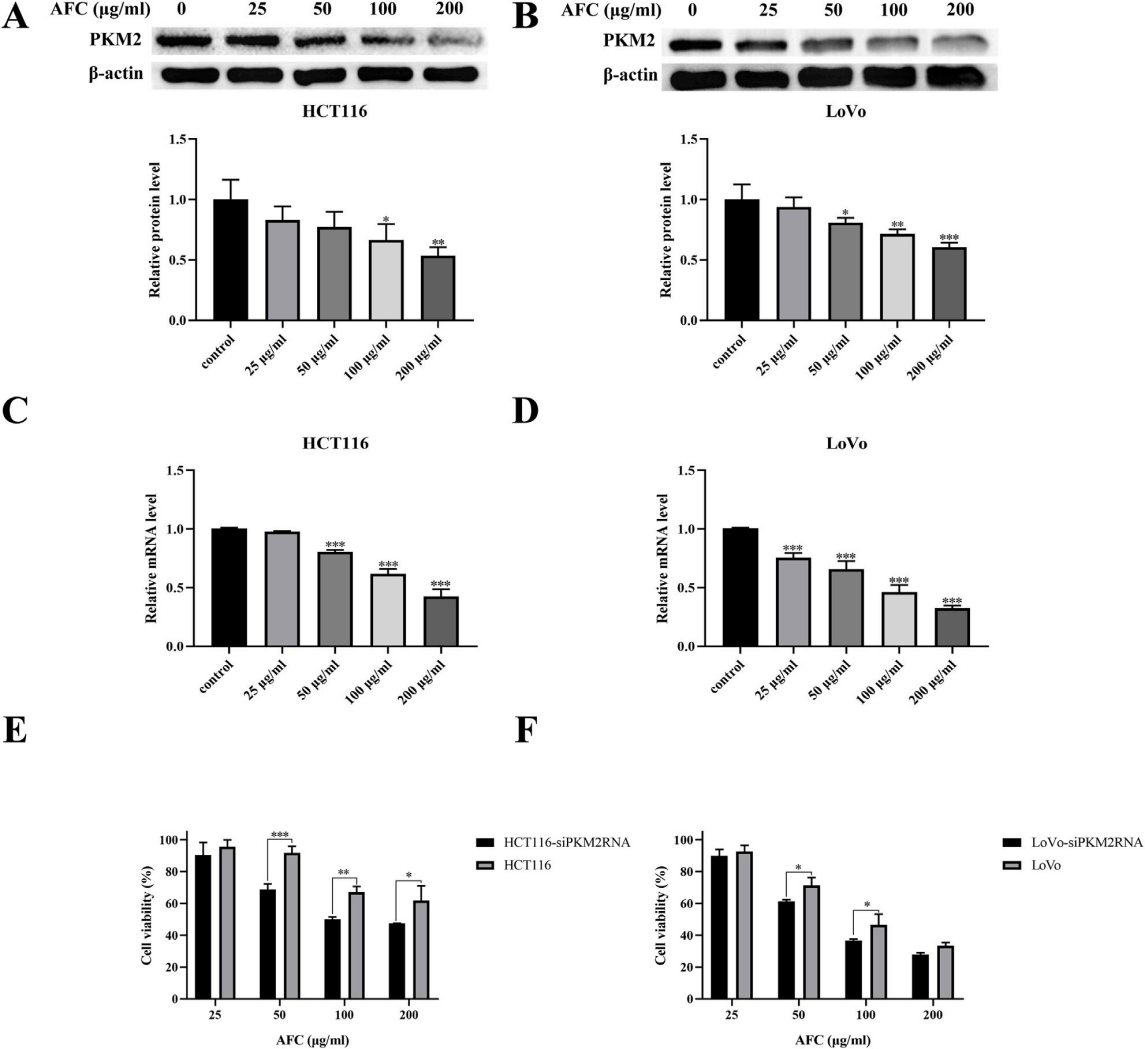
Effects of AFC on the expression of M2-type pyruvate kinase in the human colon cancer cell lines HCT116 and LoVo at the level of **(A, B)** protein based on Western blotting or **(C, D)** mRNA based on reverse transcription-quantitative PCR. **(E, F)** The viability of cancer cells in which PKM2 was knocked down (siPKM2RNA) was assessed after treatment with AFC. In all experiments, cultures were exposed to the indicated concentrations of AFC for 48 h. Data are expressed as mean ± SD of three independent experiments. *P < 0.05, **P < 0.01, and ***P < 0.001 vs vehicle control.

Nearly all types of cancer cells appear to overexpress PKM2 to promote aerobic glycolysis and provide tumor cells with a proliferative advantage over healthy tissue (Zhang et al., 2019; Huang et al., 2017; Lv et al., 2023; Huang et al., 2021). We found that AFC downregulated PKM2 in HCT116 and LoVo colon cancer cell lines in a dose-dependent manner (Figures 5A–D). In fact, cell viability was significantly reduced even further in colon cancer cells in which the PKM2 had been knocked down using siRNA (Figures 5E, F).

**FIGURE 6:**
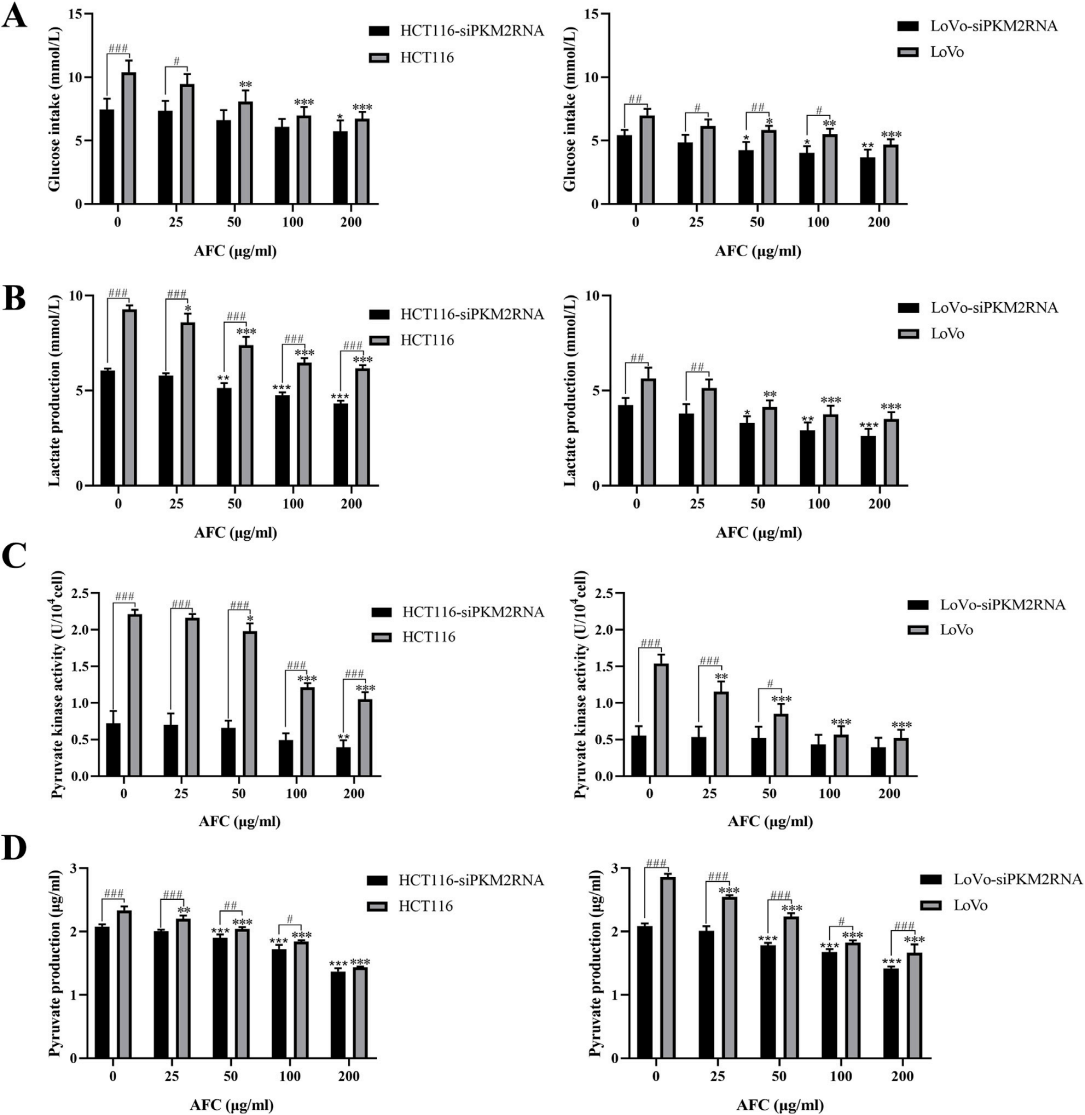
Effects of AFC on **(A)** glucose intake, **(B)** lactate production, **(C)** pyruvate kinase activity, and **(D)** pyruvate production in the human colon cancer cell lines HCT116 or LoVo in which PKM2 was knocked down (siPKM2RNA). In all experiments, cultures were exposed to the indicated concentrations of AFC for 48 h. Data are expressed as mean ± SD of three independent experiments. *P < 0.05, **P < 0.01, and ***P < 0.001 vs. the HCT116 or LoVo group (0 μg/mL); *P < 0.05, **P < 0.01, and ***P < 0.001 vs. the HCT116-siPKM2RNA (0 μg/mL) or LoVo siPKM2RNA (0 μg/mL) group; #P < 0.05, ##P < 0.01, and ###P < 0.001 (HCT116 or LoVo)-siPKM2RNA vs. the HCT116 or LoVo group.

Downregulation of PKM2 inhibited aerobic glycolysis in colon cancer cells. These cultures exhibited even lower glucose uptake (Figure 6A), lactate production (Figure 6B), pyruvate kinase activity (Figure 6C), and pyruvate production (Figure 6D) than drug-treated cells in the absence of PKM2 silencing. In addition, the results after AFC treatment suggested that AFC significantly inhibited aerobic glycolysis in colon cancer cell lines after knockdown of PKM2 in a concentration-dependent manner. The importance of PKM2 downregulation in the mechanism of AFC was confirmed by demonstrating that PKM2 knockdown on its own mimicked the effects of AFC on cell proliferation and aerobic glycolysis.

### 3.5 AFC downregulates c-myc and cyclin D1 in colon cancer cells

**FIGURE 7:**
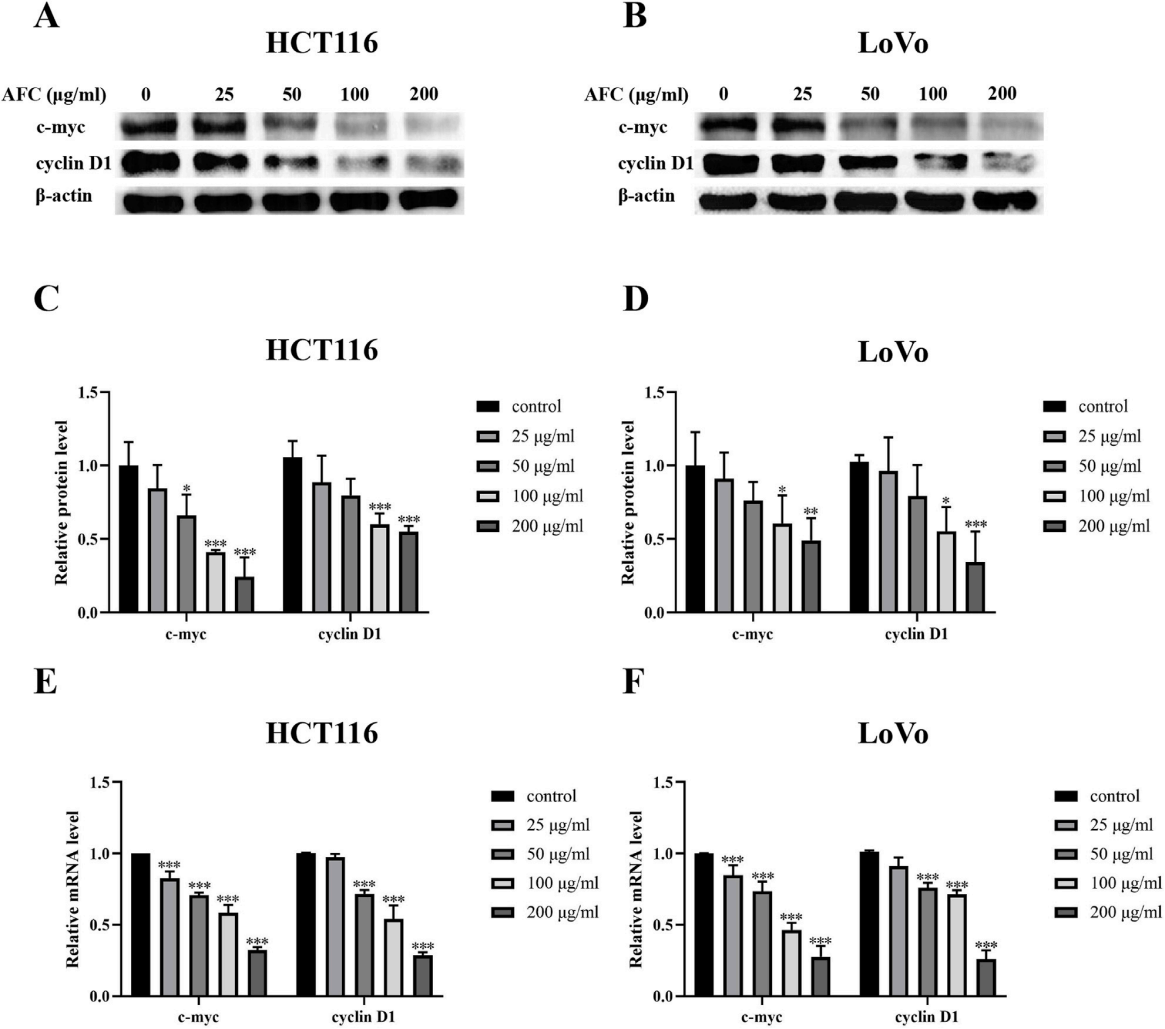
Effects of AFC on the expression of c-myc and cyclin D1 in the colon cancer cell lines HCT-116 or LoVo at the **(A–D)** protein level based on Western blotting or **(E, F)** mRNA based on reverse transcription–quantitative PCR. Data are expressed as mean ± SD of three independent experiments. *P < 0.05, **P < 0.01, and ***P < 0.001 vs. vehicle control.

Both c-myc and cyclin D1 are overexpressed in colon cancer cells (Moradifard et al., 2022; Wang et al., 2017; Musgrove et al., 2011; Santarius et al., 2010; Böckelman et al., 2012), and we found that AFC downregulated both c-myc and cyclin D1 at the protein (Figures 7A-D) and mRNA levels (Figures 7E, F) in HCT116 and LoVo cells.

### 3.6 AFC downregulates PKM2, c-myc, and cyclin D1 in colon cancer cells *in vivo* while inhibiting tumor growth

**FIGURE 8:**
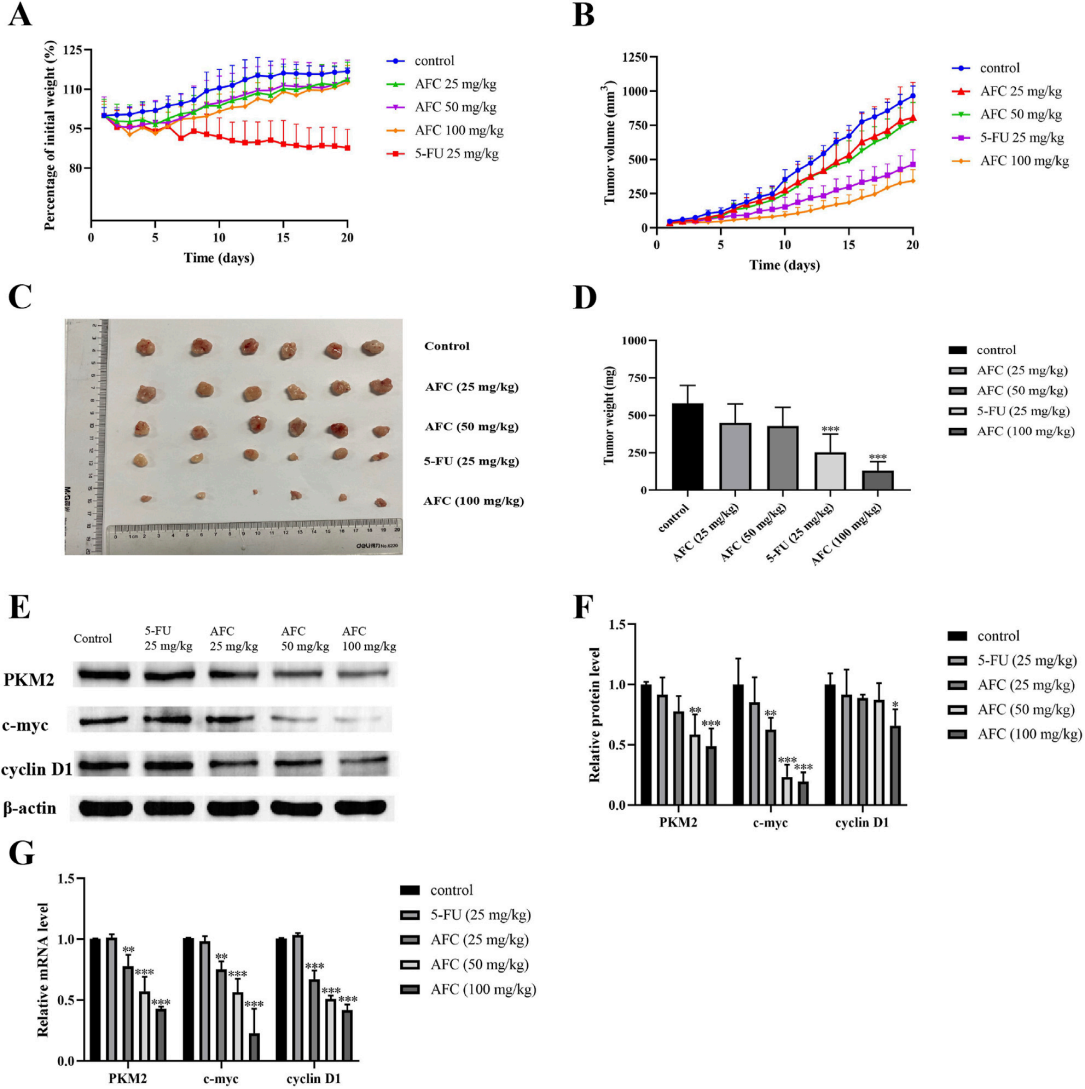
Effects of AFC on the growth of HCT116 xenografts in mice and tumor expression of PKM2, c-myc, and cyclin D1. **(A)** Body weight of animals treated in different ways, expressed as a percentage of body weight at baseline. **(B)** Tumor volume in animals treated in different ways. **(C, D)** Tumor sizes and weights at the end of the experiment. **(E, F)** Expression of PKM2, c-myc, and cyclin D1 in tumor tissues based on Western blotting. Protein levels were normalized to those of β-actin and expressed as a ratio relative to that in control cultures. **(G)** Expression of PKM2, c-myc, and cyclin D1 in tumor tissues based on reverse transcription–quantitative PCR. Levels of mRNA were normalized to those of GAPDH and expressed as a ratio relative to that in control cultures. Data are expressed as mean ± SD of three independent experiments. *P < 0.05, **P < 0.01, and ***P < 0.001 vs vehicle control.

Intraperitoneal injection of AFC inhibited the growth of HCT116 xenograft tumors in nude mice in a dose-dependent manner, such that the highest dose of 100 mg/kg was more effective than 5-FU at 25 mg/kg (Figures 8A–D). In contrast to 5-FU, none of the doses of AFC significantly reduced the body weight of animals during the experiment. AFC significantly downregulated PKM2, c-myc, and cyclin D1 in the tumors (Figures 8E–G), which is consistent with our findings *in vitro*.

Immunohistochemistry of tumor sections confirmed that AFC downregulated PKM2 to a greater extent than 5-FU did, while also downregulating the marker of tumor cell proliferation Ki67. At the same time, AFC led to a loose arrangement of tumor cells and necrotic foci full of cellular debris. The anti-tumor effects of AFC were associated with milder liver histopathology compared to 5-FU. An additional figure file shows this in more detail (Supplementary Material 1).

## 4 Discussion and conclusions

In this study, we confirm the strong ability of AFC to inhibit the growth of colon cancer cells, and we provide the first evidence that it does so by downregulating PKM2, the rate-limiting enzyme in glucose metabolism (Graziano et al., 2017; Israelsen and Vander Heiden, 2015; Wang C. et al., 2020). Simultaneous *ex vivo* and *in vivo* results also confirmed that the expression of the tumor-specific highly expressed targets c-myc and cyclin D1 was also reduced in response to AFC. Our findings provide the basis for further mechanistic investigations of AFC for its use against colon cancer and other malignancies. Our work also extends the number of contexts in cancer in which PKM2 promotes the Warburg effect (de Padua et al., 2017; Zhao et al., 2022).

Pyruvate kinase catalyzes the last step of glycolysis, regardless of whether oxygen is present or not (El-Far et al., 2022; Zhu et al., 2021), and PKM2 is overexpressed in cancer cells (Chaneton and Gottlieb, 2012; Wang X. et al., 2020; Kim et al., 2019). Similarly, c-myc is upregulated in cancer cells, and it induces the expression of genes that drive cell proliferation and aerobic glycolysis to promote the Warburg effect (Patel et al., 2004; Fatma et al., 2022; Wu et al., 2020). Cyclin D1 is also upregulated in many cancers, including most cases of colorectal cancer, and it promotes the proliferation and metastasis of tumor cells (Li et al., 2022; Qi et al., 2019). The three proteins even interact with one another to promote tumor proliferation in non-metabolic nuclear transcription pathways: PKM2 can translocate from the cytoplasm into the nucleus, where it induces histone phosphorylation that turns on the genes encoding c-myc and cyclin D1 (Venneti and Thompson, 2013; Yang et al., 2012; Zheng et al., 2020; Yang et al., 2011; Lu, 2012; Wu et al., 2023). All these studies will be the theoretical basis for further molecular mechanistic studies.

Our findings justify further investigation of the effects of AFC on non-metabolic pathways in colon cancer, starting with pathways regulated by PKM2. Our observations that AFC may be even more effective than 5-FU against colon cancer while inducing milder liver toxicity argue for the continued investigation of AFC’s therapeutic mechanisms. Future work should explore how it downregulates pyruvate kinase in the first place, and the intrinsic mechanism between the downregulation of PKM2 and the downregulation of c-myc and cyclin D1.

Through *in vitro* and *in vivo* experiments, we demonstrated that AFC could affect aerobic glycolysis in colon cancer cells against colon cancer by downregulating PKM2. We also found that AFC not only downregulated PKM2 in colon cancer models but also downregulated the expression of the tumor-specific, highly expressed targets c-myc and cyclin D1. PKM2 functions as an upstream regulatory protein in the nucleus and triggers the transcription of the c-myc and cyclin D1 genes 67 via H3-T11. This suggests that our next research direction should be to verify the intrinsic relationship and mechanism of PKM2 with c-myc and cyclin D1 in non-metabolic pathways. In addition, based on the role of aerobic glycolysis in tumor development, we will also gradually expand the target of AFC to include other key enzymes involved in aerobic glycolysis.

## Data Availability

The raw data supporting the conclusions of this article will be made available by the authors, without undue reservation.

## Abbreviations

AFC, active fraction from clove; CRC, colorectal cancer; DMEM, Dulbecco’s modified Eagle medium; 5-FU, 5-fluorouracil; PKM2, M2-type pyruvate kinase; RT-qPCR, reverse transcription quantitative polymerase chain reaction; siRNA, small interfering RNA.
